# Submerged Fermentation of *Rhizopus* sp. for l‑asparaginase Production in Lymphoma Therapy

**DOI:** 10.1021/acsomega.5c06775

**Published:** 2025-10-17

**Authors:** Nicoly Subtil de Oliveira, Romeu Cassiano Pucci da Silva Ramos, Pedro Andrade de Souza, Vinicius Chaves Mesacasa, Luiz Fernando Bianchini, Edvaldo Antonio Ribeiro Rosa

**Affiliations:** † Xenobiotics Research Unit, 28100 Pontifícia Universidade Católica do Paraná, Curitiba 80215-901, Brazil; ‡ Graduate Program in Animal Sciences, Pontifícia Universidade Católica do Paraná, Curitiba 80215-901, Brazil; § Graduate Program on Dentistry, Pontifícia Universidade Católica do Paraná, Curitiba 80215-901, Brazil

## Abstract

l-asparaginase (L-ASNase) has garnered considerable interest in lymphoma therapy due to its mechanism of action, which involves depleting the availability of circulating l-asparagine, a crucial nutrient for malignant cells. Given the notable side effects of commercially available bacterial L-ASNase and the existing challenges in large-scale industrial production, this study focused on identifying and optimizing novel fungal sources for the enzyme. The research methodology involved initial strain screening, development and optimization of a bioreactor system, and subsequent evaluation of enzyme production within a bioprocess. A comprehensive screening of various fungal strains revealed that *Rhizopus* sp. UPX271019 exhibited the highest specific activity, indicating its significant potential for therapeutic and industrial applications. The study investigated the performance of various bioreactor configurations. A fixed-bed airlift hybrid bioreactor (FB-ALR), operated with an aeration rate of 0.75 vvm, demonstrated superior hydrodynamic and mass transfer performance. A critical observation was the significant role of biofilm formation by *Rhizopus* sp. UPX271019 within the FB-ALR, which was instrumental in achieving substantial L-ASNase production. The bioprocess yielded an average enzyme activity of 2475 ± 701 U, highlighting the system’s effectiveness. These results suggest that the FB-ALR operating with such a strain grown as biofilm is a highly suitable system for future industrial scale-up.

## Graphical abstract

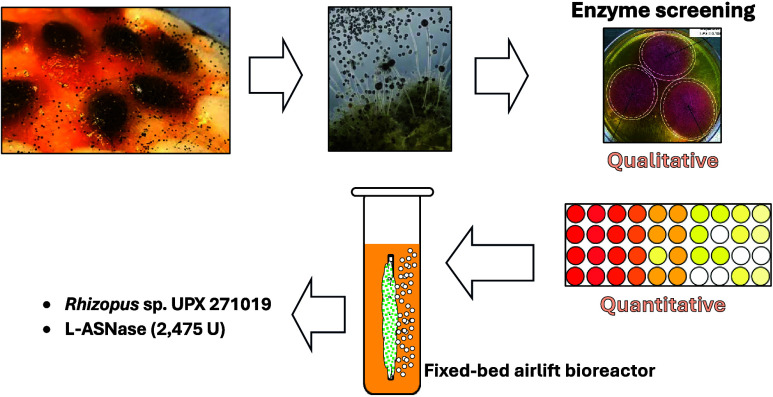

## Introduction

l-asparagine is an essential amino acid for protein biosynthesis that can be produced in living cells by asparagine synthetase.^1^ Human and animal tumor cells require large amounts of l-asparagine for their rapid growth; however, they do not synthesize it, instead consuming the low concentrations available in neoplastic cells^2^ or the amino acid from the diet.^3^

l-asparaginase (L-ASNase; l-asparagine amide hydrolase, E.C. 3.5.1.1) is an enzyme belonging to an amidase group that hydrolyzes free l-asparagine into aspartic acid and ammonia. In this context, L-ASNase has been used to reduce the supply of l-asparagine in the blood, selectively affecting neoplastic cells.^4^

Most studies involving L-ASNase have explored its use in the human clinic, with relatively little investigation into animal patients remaining focused on dogs and cats.^5,6^ The data from these investigations are promising, leading the American Veterinary Medical Association (AVMA) to formally petition the Food and Drug Administration (FDA) to approve the inclusion of this enzyme on the list of approved ingredients for formulations designated for these species.^7^

However, commercially available L-ASNase is of bacterial origin (derived from *Escherichia coli* or *Erwinia* spp.) and has a toxicity profile that may involve acute hypersensitivity and other iatrogenic complications in humans and animals.^8^

Due to these adverse effects, the search for other sources of L-ASNase and forms of production has increased. Eukaryotic microorganisms, such as filamentous fungi, may present fewer adverse effects due to their greater evolutionary proximity to animal genetic material.^9–12^

On the other hand, the bioprocesses to obtain L-ASNase from these organisms need to be better established since filamentous fungi have macromorphologies that make it unfeasible to use bioreactors typically used in upstream stages involving bacteria or yeast.^13^

Research into methods for obtaining L-ASNase of fungal origin has gained interest in the last two decades,^14–17^ with promising experimental results.^18^ However, to our knowledge, only bacterial variants of the enzyme have been commercialized for clinical use, whether for human or animal use. This scenario partly arises from the lack of standardization of bioprocesses that make producing fungal enzyme versions viable. These factors led us to conduct this study.

For these reasons, this study requested a fast-growing filamentous fungus that produced reasonable amounts of L-ASNase but did not produce l-glutaminase (L-GLNase, which converts l-glutamine into the undesirable l-glutamic acid, an excitatory neurotransmitter associated with pain) and, in parallel, developed and optimized a bioreactor to produce fungal L-ASNase. The study focused solely on upstream stages.

## Material and Methods

### Qualitative Screening of L-ASNase-Producing Strains

Twenty-six strains from nine genera were evaluated for their possible ability to produce L-ASNase (Table 1).

**Table 1:** Fungal Strains Submitted to Qualitative Screening for L-ASNase Production

strain	origin
*Arthrinium xenocordella* AV23	coconut endophyte
*Aspergillus niger* 3438	industry strain
*Aspergillus niger* CH	industry strain
*Aspergillus niger* UPX-Aniger01	onion decomposer
*Beauveria bassiana* C4Bio	entomopathogen
*Cunninghamella blakesleeana* DSM1906	unknown
*Cunninghamella echinulata* DSM1905	unknown
*Cunninghamella elegans* DSM1908	flax endophyte
*Cunninghamella elegans* DSM63299	unknown
*Cunninghamella elegans* DSM8217	estuary silt
*Diaporthe arengae*	coconut endophyte
*Diaporthe endophytica* PB80	coconut endophyte
*Guignardia mangiferae* AA118	coconut endophyte
*Penicillium* sp. 3P1	soil
*Penicillium citrinum* PB38	coconut endophyte
*Penicillium* sp. UPX-Pen01	citrus decomposer
*Purpureocillium lilacinum* PB41	coconut endophyte
*Rhizopus* sp. UPX021019	strawberry decomposer
*Rhizopus* sp. UPX081019	strawberry decomposer
*Rhizopus* sp. UPX240919	strawberry decomposer
*Rhizopus* sp. UPX250919	strawberry decomposer
*Rhizopus* sp. UPX271019	passion fruit decomposer
*Rhizopus* sp. UPX280919C	strawberry decomposer
*Rhizopus* sp. UPX291019	passion fruit decomposer
*Rhizopus* sp. UPX300919	strawberry decomposer
*Rhizopus* sp. UPX311019	passion fruit decomposer

Petri dishes containing Sabouraud Dextrose Agar (SDA) were seeded with different strains and kept at 28 °C for 5 days. Five-millimeter discs of mycelia were generated with sterile punches. One disc per plate was transferred to plates containing 15 mL of CDM-agar [2 g L^–1^ glucose, 10 g L^–1^ l-asparagine, 1.52 g L^–1^ KH_2_PO_4_, 0.52 g L^–1^ MgSO_4_·7H_2_O, 0.52 g L^–1^ KCl, 0.001 g L^–1^ CuNO_3_·3H_2_O, 0.001 g L^–1^ ZnSO_4_·7H_2_O, 0.001 g L^–1^ FeSO_4_·7H_2_O, 2% (w/v) agar–agar, 0.009% (w/v) phenol red; pH 6.2]. Plates with CDM-agar without l-asparagine served as controls. The plates were incubated at 28 °C for 120 h. Reddish zones formed around the colonies indicated possible production of L-ASNase. Plates with modified CDM-agar, containing 10 g L^–1^ l-glutamine instead of l-asparagine, were used to analyze the potential production of L-GLNase.

The diameters of colonies and halos were measured with a digital caliper in the N–S, E-W, NE-SW, and NW-SE orientations. The halo areas (HA) were calculated and divided by the means of those of the colonies (CA). The results were then subtracted from one, which indicates that the higher the value, the greater the hydrolytic activity (eq 1).

1 enzymaticactivity=1−(HA/CA)

The assays were conducted in triplicate with three colonies per plate, totaling nine independent individual measurements. Those with the highest L-ASNase and zero L-GLNase production were selected for further assays.

### Quantitative Screening of L-ASNase-Producing Strains

The selected strains were cultivated in SDA for 7 days. After incubation, 2 mL aliquots of 0.05% (v/v) Tween 20 were added, and the plates were gently rocked to deliver conidia. Aliquots of 1 mL were collected and transferred to 250 mL Erlenmeyer flasks containing 50 mL of CDM-broth (2 g L^–1^ glucose, 10 g L^–1^ l-asparagine, 1.52 g L^–1^ KH_2_PO_4_, 0.52 g L^–1^ MgSO_4_·7H_2_O, 0.52 g L^–1^ KCl, 0.001 g L^–1^ CuNO_3_·3H_2_O, 0.001 g L^–1^ ZnSO_4_·7H_2_O, 0.001 g L^–1^ FeSO_4_·7H_2_O; pH 6.2 and no indicator).

Cultures were maintained at 30 °C and 120 rpm for 120 h. Biomasses and supernatants were separated by centrifugation (2000*g*; 10 min; 4 °C). L-ASNase activity in supernatants was evaluated using a method for microplate quantifications by estimating the amount of ammonia released in the reaction using Nessler’s reagent.^19^

Reactions were initiated by adding 50 μL of supernatant to 200 μL of 40 mM l-asparagine and 50 μL of 50 mM Tris-HCl buffer (pH 7.2) and incubating at 35 °C for 60 min. The reactions were stopped by adding 50 μL of 1.5 M trichloroacetic acid (TCA). Aliquots of 25 μL of the supernatants were added to 187.5 μL of distilled water and 37.5 μL of Nessler’s reagent. Readings were performed in 96-well flat-bottom microplates in a microplate reader TP-Reader (ThermoPlate Inc., Nanshan District, Shenzhen, China), at 425 nm.

The amounts of ammonia released by the samples were calculated from a standard curve of ammonium sulfate (68.2 to 8727.2 μmol L^–1^). The enzymatic activity of each strain was arbitrarily assigned as the amount of enzyme that catalyzes the conversion of 1 μmol of l-asparagine per minute at 35 °C under the assay conditions and expressed in enzymatic units (eq 2). In the equation, 0.35 is the total volume in the first assay step (mL), 0.025 is the fraction volume (mL) used for nesslerization, 60 is the assay time (min), and 0.05 is the volume of sample used in the assay.

2 $$U=\left[\left({\mathrm{\mu mol}}_{\mathrm{NH}3}\times 0.35\right)/\left(0.025\times 60\times 0.05\right)\right]$$

Biomasses were obtained by gravimetry. The mycelia were filtered on filter paper (PF) with a previously determined mass, followed by drying at 80 °C for 24 h. The filter paper + mycelium (PFM) set was weighed, and the mass of the PF was subtracted (eq 3).

3 drymass=PFM−PF

To determine the enzyme production as a function of the fungal biomass produced in the bioprocess and obtain the specific enzymatic activity, the enzymatic activity determined above was divided by the dry fungal biomass (eq 4).

4 $${Ug}^{-1}=\left[\left({\mathrm{\mu mol}}_{\mathrm{NH}3}\times 0.35\right)/\left(0.025\times 60\times 0.05\right)\right]/\mathrm{biomass}\left(g\right)$$

### Design and Construction of the Bioreactor

A split-rectangle-internal loop airlift bioreactor (SRILAB) was built in AISI304 stainless steel with a wall thickness of 2 mm, a square section of 75 mm (Ø), and a height of 1000 mm (h). The working volume is 4.00 L, with an operational geometric ratio of 5:1 (Figure 1). Four inspection windows were installed throughout its structure. On the riser, there is one at the base (50 mm) for inspecting the aeration system (sparger), one at a height of 400 mm to check the upward flow of bubbles, and one at 750 mm for foam monitoring. The downcomer is 400 mm tall for monitoring the bubbles’ downward flow.

**Figure 1:**
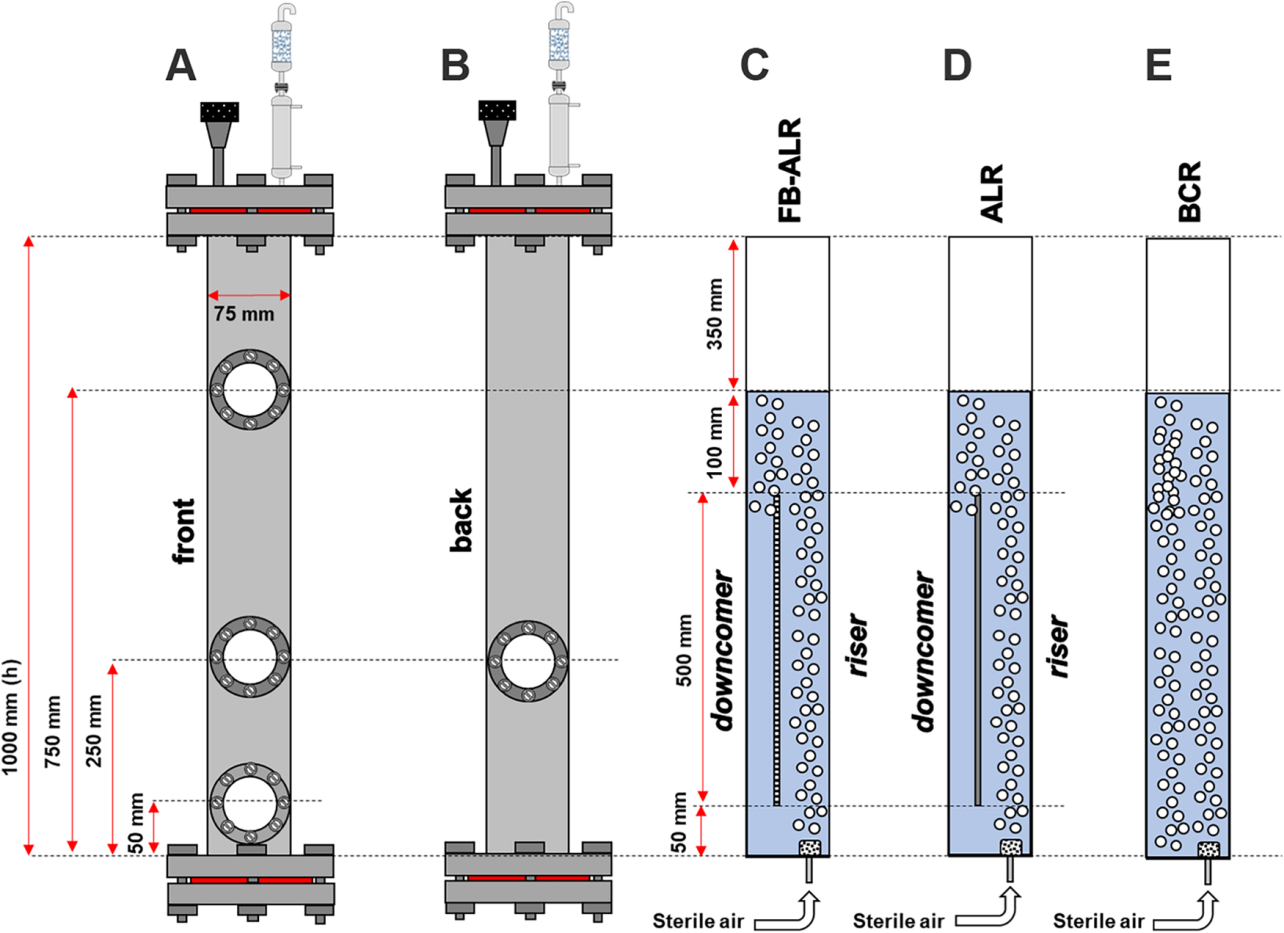
SRILAB structure. (A) front external view; (B) back external view; (C) internal view operating as FB-ALR; (D) internal view operating as ALR; (E) internal view operating as BCR.

A 320 × 75 mm baffle made with 2 mm thick AISI304 stainless steel was adapted 50 mm from the bottom and 46.35 mm from one of the walls so that it provides a riser-to-downcomer (A_r_/A_d_) partition that follows the golden ratio φ = (1 + √5)/2 = 1.618 (Figure 2).^20^ The volumes occupied by the riser and downcomer are 1.74 and 1.06 L, respectively. Similarly, a reticulated baffle of 320 × 75 mm AISI304 stainless steel #30 mesh (0.25 mm wire, 0.60 mm opening) was adapted as a scaffold for fungal biofilm formation. After being completely colonized, it works as a typical baffle.^21^

**Figure 2:**
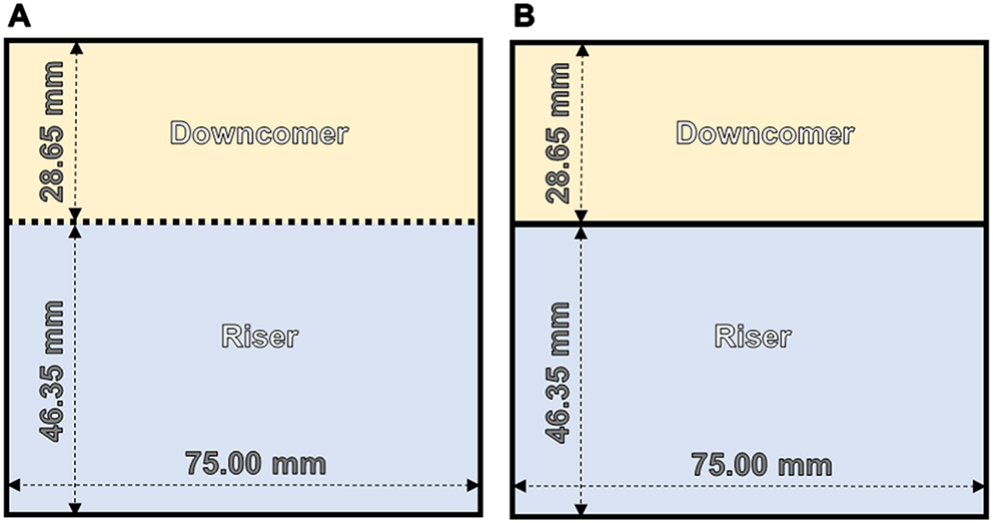
SRILAB internal geometries when operating as FB-ALR (A) and ALR (B).

At the top, connections and accesses for adapting electrodes, thermometers, and probes compatible with the sensors’ diameters were provided.

An oil-free dental air pump provided the air supply. Aeration occurs using a sparger made from sintered AISI316L stainless steel with 2 μm Ø pores. Membrane filters of 0.22 μm Ø placed in the air pipeline guaranteed the sterility of the gas admitted into the system.

### Hydrodynamic Parameters

All tests were conducted at a constant temperature of 25 °C and 678 ± 10 mmHg, with the bioreactors operating as bubble column (BCR; i.e., without any baffle), airlift (ALR; i.e., with solid baffle), and airlift-fixed bed (FB-ALR; i.e., with reticulated baffle).

#### Fluid

The tap water supply from Curitiba (Brazil) (CWB-tw) (μ = 0.89 mPa·S @ 25 °C) was used as the Newtonian fluid.

#### Airflow

Arbitrarily, the SRILAB was defined as operating with four flow regimes (0.25 vvm/1.0 L min^–1^; 0.50 vvm/2.00 L min^–1^; 0.75 vvm/3.00 L min^–1^; 1.00 vvm/4.00 L min^–1^).

#### Linear Gas Velocity (*U*_G_)

It was determined by dividing the volumetric air flow (m^3^ min^–1^) by the riser area (m^2^). *U*_G_ values were expressed as m s^–1^.

#### Liquid Linear Velocity (*U*_L_)

The SRILAB was filled with its working volume (4 L). Air was admitted into the bioreactor in four different flows for 30 s. Five milliliters of 1% (w/v) methylene blue were carefully dispensed onto the top of the downcomer region adjacent to the wall. The mixtures were monitored for the time needed to reach the different inspection windows. The time intervals were determined with a digital chronograph, and the interpolation of the various times generated the *U*_L_, which was expressed in m s^–1^.

#### Mixing Time (*t*_M_)

The time required for the dye front to reach the surface of the liquid was assumed as t_M_ and was expressed in seconds (s). For sequential repetitions, the SRILAB was drained, washed, and filled with a new load of water.

#### Global Gas Retention (ε_G_)

A U-shaped pressure gauge is fitted to the top of the riser and the downcomer. The bioreactor was filled with CWB-tw and subjected to the aeration regimes described above. After 30 s of stabilization, the column’s linear displacement (*m*) was used to determine the ε_G_ values using eq 5. *V*_i_ is the initial volume, and *V*_f_ is the final volume.

5 $$\varepsilon_{G}=V_{i}/\left(V_{i}+V_{f}\right)$$

#### Hydrostatic Pressure (*P*_H_)

The liquid phase oscillations were converted into linear height, and using eq 6, it was possible to establish the existing pressure after 30 s of gas admission. The components of the equation are *P*_H_ = pressure (MPa), pm = 1000 kg m^–3^, *h* = δ height (m), g = 9.81 m s^–2^, and *p*_atm_ (Curitiba) = 1.024 MPa.

6 $$P_{H}=\left(p_{m}\times h\times g\right)+P_{\mathrm{atm}}$$

#### Volumetric Oxygen Transfer Coefficient (*k*_L_a)

The SRILAB was filled to the workload with CWB-tw. Nitrogen was sparged into the system at 4 L min^–1^ using a three-way valve system. The drop in dissolved oxygen (DO) concentration was monitored by a DO-5509 oximeter (Lutron Electron. Enterp. Co. Ltd., Taiwan). After oxygen removal (DO ≤ 0.3 mg L^–1^), the nitrogen valve was turned off, and the atmospheric air was admitted with four flow regimes (0.25 vvm (1.25 L min^–1^); 0.50 vvm (2.5 L min^–1^); 0.75 vvm (3.75 L min^–1^); 1.00 vvm (5.00 L min^–1^)). DO concentrations were monitored until uniform saturation (typically ∼8.1 mg L^–1^). OD increments were plotted against time (s^–1^). Equation 7 was used to establish the different *k*_L_a and its components are *k*_L_, oxygen transfer coefficient (L t^–1^); a is the area of the gas/liquid interface (L L^–3^); *k*_L_a, oxygen transfer coefficient (1 t^–1^); and C*, oxygen concentration at saturation (m L^–3^) (approximately 7 mg L^–1^ at 25 °C and 1 atm). In mass transfer experiments, it is assumed that the *k*_L_a value is constant for the entire bioreactor.

7 $$-\ln\left(1-\frac{C-C_{0}}{C^{*}-C}\right)=k_{L}a\left(t-t_{0}\right)$$

### Submerged Fermentation in SRILAB

The SRILAB operated on the FB-ALR system, and the stainless steel screen was a baffle. Recently, it was demonstrated that such a bioreactor system is the most profitable for fungal L-ASNase.^22^ It was filled with 4 L of modified CDM broth (2 g L^–1^ l-asparagine; pH 6.0) and that was thermally disinfected (100 °C) in place for 20 min using a 7000 W electric resistance heater.^23^ Contamination was monitored at the end of the process, and no contaminant growth (even for bacterial spores) was noticed. After cooling, 30 mL of inoculum were added. This inoculum was produced by crushing fungal mycelium in a Tenbroek homogenizer for 5 min. The suspension of mycelial fragments was corrected to a turbidity close to McFarland tube #5.

Aeration of 0.75 vvm was adopted, and the SRILAB was installed at 25 ± 1 °C. The fungal biomass production took place for 72 h, and then l-asparagine (10 g L^–1^) was added for enzymatic induction. From this point, L-ASNase production lasted 120 h.

To calculate the specific enzyme activity, enzyme activity^19^ and protein concentrations^24^ were determined.

### Statistical Analysis

The Tukey HSD test was applied to the results of the different screening assays. The results obtained for the different bioreactors were subjected to verification of normality of distribution using the Kolmogorov–Smirnov test. As the dependent variables presented a normal distribution, one-way ANOVA was performed. When there was an indication of a difference between at least two bioreactors, a 2-to-2 comparison was performed using the Tukey HSD test for homogeneous variances. The power of the test was 94.97%, and the significance level adopted in all tests was 5% (*p* < 0.05).

## Results and Discussion

### Qualitative Screening of L-ASNase-Producing Strains

Measurements of the areas of colonies and halos in the CDM-agar medium were carried out to preliminarily semiquantify the production of L-ASNase (Table 2). It is essential to state that the L-ASNase here prospected is constitutively produced from the endogenous gene(s) in the genome of these species, not produced after any genetic manipulation.

**Table 2:** Colonies Areas (CA) and Hydrolysis Halos (HA) for L-ASNase and L-GLNase Obtained during Screening Assays^a^

	L-ASNase				L-GLNase			
strain	CA (mm^2^)	HA (mm^2^)	HA/CA	1-Pz^b^	CA (mm^2^)	HA (mm^2^)	HA/CA	1-Pz
*Arthrinium xenocordella* AV23	180.26 ± 24.03	0.00 ± 0.00						
*Aspergillus niger* UPX-Aniger01	128.27 ± 15.31	0.00 ± 0.00						
*Beauveria bassiana* C4Bio	80.43 ± 36.75	0.00 ± 0.00						
*Diaporthe arengae*	253.90 ± 61.96	0.00 ± 0.00	nc^c^	nc	nc	nc	nc	nc
*Guignardia mangiferae* AA118	56.74 ± 10.60	0.00 ± 0.00						
*Penicillium* sp. UPX-Pen01	192.85 ± 46.88	0.00 ± 0.00						
*Rhizopus* sp. UPX021019	844.46 ± 345.92	0.00 ± 0.00						
*Aspergillus niger* 3438	381.19 ± 244.23	416.44 ± 250.38	1.09	0.08	41.82 ± 7.03	258.20 ± 98.99	6.17	0.83
*Aspergillus niger* CH	69.07 ±11.55	79.68 ± 12.28	1.15	0.13	38.74 ± 5.87	123.29 ± 15.09	3.18	0.68
*Diaporthe endophytica* PB80	93.02 ± 44.28	352.60 ± 233.89	3.79	0.73	124.77 ± 28.98	141.92 ± 28.31	1.13	0.12
*Penicilium* sp. 3P1	42.88 ± 17.08	318.89 ± 233.52	7.43	0.86	42.00 ± 9.60	65.77 ± 24.19	1.56	0.36
*Penicillium citrinum* PB38	64.73 ± 22.90	242.92 ± 161.72	3.75	0.78	57.31 ± 10.14	157.89 ± 77.05	2.75	0.63
*Purpureocillium lilacinum* PB41	66.54 ± 6.53	244.78 ± 49.53	3.67	0.75	64.13 ± 9.53	396.43 ± 105.57	6.18	0.83
*Cunninghamella blakesleeana* DSM1906	68.20 ± 12.21	262.12 ± 109.04	3.84	0.74	583.20 ± 20.88	0.00 ± 0.00		
*Cunninghamella echinulata* DSM1905	125.18 ± 48.24	146.02 ± 56.26	1.16	0.14	223.48 ± 12.31	0.00 ± 0.00		
*Cunninghamella elegans* DSM1908	390.74 ± 47.13	878.78 ± 119.13	2.24	0.55	360.01 ± 5.69	0.00 ± 0.00		
*Cunninghamella elegans* DSM63299	722.38 ±168.62	1241.28 ± 328.22	1.71	0.41	705.91 ± 32.28	0.00 ± 0.00		
*Cunninghamella elegans* DSM8217	528.89 ± 33.37	1325.70 ± 69.09	2.50	0.60	544.49 ± 12.15	0.00 ± 0.00		
*Rhizopus* sp. UPX240919	2085.36 ± 887.31	6370.23 ± 207.51	3.05	0.67	2234.85 ± 696.28	0.00 ± 0.00		
*Rhizopus* sp. UPX250919	1402.03 ± 331.29	6361.74 ± 151.21	4.54	0.78	1421.34 ± 16.06	0.00 ± 0.00	nc	nc
*Rhizopus* sp. UPX280919C	2274.19 ± 846.94	6381.63 ± 136.67	2.81	0.64	2131.09 ± 936.18	0.00 ± 0.00		
*Rhizopus* sp. UPX300919	1826.12 ± 620.01	6321.46 ± 163.54	3.46	0.71	1988.95 ± 762.83	0.00 ± 0.00		
*Rhizopus* sp. UPX081019	1709.24 ± 77.55	6300.03 ± 174.96	3.69	0.73	1675.22 ± 46.32	0.00 ± 0.00		
*Rhizopus* sp. UPX271019	1306.71 ± 182.44	6315.04 ± 320.20	4.83	0.79	1106.27 ± 386.80	0.00 ± 0.00		
*Rhizopus* sp. UPX291019	1515.99 ± 644.73	6305.15 ± 198.52	4.16	0.76	2043.11 ± 596.18	0.00 ± 0.00		
*Rhizopus* sp. UPX311019	1537.08 ± 213.40	6215.30 ± 231.58	4.04	0.75	1402.36 ± 247.16	0.00 ± 0.00		

a Nine replicates were carried out per strain.

b Pz is the value obtained by dividing CA by HA; the higher the 1-Pz value higher the enzyme activity.

c nc indicates no enzyme activity.

It was possible to observe that the strains *C. blaskesleeana* DSM1906, *C. echinulata* DSM1905, *C. elegans* DSM 1908, *C. elegans* DSM63299, *C. elegans* DSM8217, and *Rhizopus* sp. UPX240919, *Rhizopus* sp. UPX250919, *Rhizopus* sp. UPX280919C, *Rhizopus* sp. UPX300919, *Rhizopus* sp. UPX081019, *Rhizopus* sp. UPX271019, *Rhizopus* sp. UPX291019, and *Rhizopus* sp. UPX311019 generated the most promising readings because it produced more L-ASNase and presented a null index for L-GLNase production. L-GLNase is an undesirable byproduct.

On the other hand, the assay made it possible to eliminate strains that produced L-GLNase or those that showed insufficient levels of L-ASNase production, such as the strains *Arthrinium xenocordella* AV23, *Aspergillus niger* UPX-Aniger01, *A. niger* 3438, *A. niger* CH, *Beauveria bassiana* C4Bio, *Diaporthe endophytica* PB80, *D. arengae*, *Guignardia mangiferae* AA118, *Penicillium* sp. UPX-Pen01, *Penicillium* sp. 3P1, *P. citrinum* PB38, *Purpureocillium lilacinum* PB41, and *Rhizopus* sp. UPX021019.

### Quantitative Screening of L-ASNase-Producing Strains

Of the preselected strains, among the L-ASNase-positive *Cunninghamella* spp., *C. echinulata* DSM1905 was the best producer (1.490 U), followed by *C. elegans* DSM8217 (1.310 U), *C. blaskesleeana* DSM1906, *C. elegans* DSM1908, and *C. elegans* DSM63299, which did not show variations in enzymatic activity, with 0.963, 1.037, and 1.098 U, respectively (Figure 3). However, the specific activity results, which translate the enzyme production capabilities per amount of biomass, were unsatisfactory, i.e., the enzyme was produced requiring a relatively large amount of biomass.

**Figure 3:**
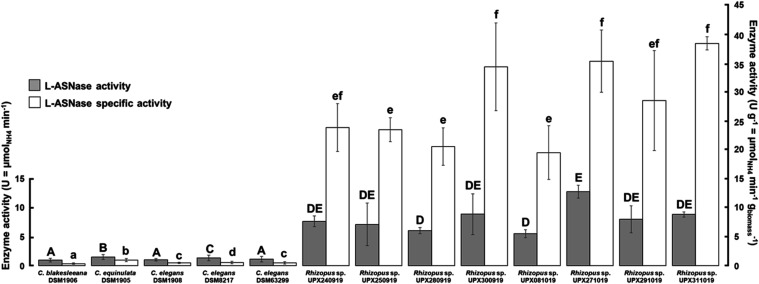
L-ASNase activity of selected strains. Different uppercase letters above gray bars denote differences (*p* ≤ 0.05) in the L-ASNase activity among the 13 strains enrolled in this study stage. Different lowercase letters above white bars denote differences (*p* ≤ 0.05) in the L-ASNase specific activity among the 13 strains enrolled in this study stage. Values were obtained for nine independent individual measurements.

In turn, the strains of *Rhizopus* spp. screened as L-ASNase-positive presented the best results in CDM-broth regarding enzyme production and specific activity values.

The screening aimed to prospect fungal strains that produced L-ASNase without L-GLNase activity. As expected, more than one strain led to good enzyme production, which was between 5.46 and 12.64 U. This last result is very close to that obtained by Ashok and Kumar,^25^ who reported preliminary enzymatic activity for *Rhizopus microsporus* IBBL-2 of the order of 12.68 U, and which was considered satisfactory. This similarity of results reveals that the strains tested in this study showed promising results for enzymatic activity, especially regarding the amount of biomass produced. Three of the tested strains generated specific enzymatic activity values greater than 30 U, and strain UPX271019 was chosen for SRILAB testing as it presented the highest enzyme activity.

As no enzyme purification was carried out at this stage, it is possible that such a measurement of protein, together with other impurities, is inaccurate, leading to an underestimation of the actual values. The idea presented here was to compare and screen those strains with better L-ASNase-producing ability from an upstream perspective. Our group is now centering efforts to develop downstream strategies to separate, concentrate, and purify L-ASNase.

The fact that *Rhizopus* was the genus that produced the most L-ASNase among the different ones listed in the study is quite interesting, as its species are a long time known to proliferate very well;^26^ several of them are widely used in the production of fermented foods;^27^ and the production of mycotoxins is restricted to a few strains, which are easily screenable and preventable.^28^

### Hydrodynamic Parameters of the Bioreactor

The production of L-ASNase by filamentous fungi in bioreactors remains poorly explored. Our proposal to use pneumatically agitated bioreactors rather than mechanically agitated ones is based on compromising the integrity of the arrangements and fungal cells when operating at high-power inputs.^29^

To understand which pneumatically agitated bioreactor would best favor fungal growth, we conducted a series of hydrodynamic tests for different gas flows, shown in Table 3.

**Table 3:** Hydrodynamic and Volumetric Mass Transference Parameters for the Different Bioreactors Operating According to the Aeration Regimens^*^

airflow (vvm)	bioreactor system	*U*_G_ (m s^‑1^)	*U*_L_ (m s^‑1^)	*t*_M_ (s)	ε_G_ (−)	*P*_H_ (MPa)	*k*_L_a (s^–1^)
0.25	BCR	0.222	0.141^a^^**^	5.137^a^	0.720^a^	0.1095^a^	0.0156^a^
	ALR	0.360	0.091^b^	11.727^b^	0.723^a^	0.1095^a^	0.0252^b^
	FB-ALR	0.360	0.062^c^	7.950^c^	0.725^a^	0.1095^a^	0.0274^c^
0.50	BCR	0.444	0.151^a^	7.510^b^	0.725^a^	0.1095^a^	0.0673^d^
	ALR	0.720	0.097^b^	14.567^d^	0.725^a^	0.1096^a^	0.0673^d^
	FB-ALR	0.720	0.081^b^	9.067^bc^	0.727^a^	0.1097^a^	0.0720^e^
0.75	BCR	0.666	0.212^d^	7.873^b^	0.734^ab^	0.1096^a^	0.0770^f^
	ALR	1.080	0.092^b^	10.331^c^	0.747^b^	0.1097^a^	0.0872^g^
	FB-ALR	1.080	0.085^b^	8.770^b^	0.747^b^	0.1099^a^	0.0873^g^
1.00	BCR	0.888	0.287^e^	6.707^ab^	0.755^b^	0.1096^a^	0.0766^f^
	ALR	1.440	0.090^b^	8.033^b^	0.767^c^	0.1098^a^	0.0877^g^
	FB-ALR	1.440	0.113^b^	8.256^b^	0.783^d^	0.1101^a^	0.0926^g^

^*^The results were obtained using tap water supply from Curitiba, Brazil (μ = 0.89 mPa·S @ 25 °C).

^**^Different superscript letters indicate differences in the same row (*p* < 0.05).

For the *U*_G_ parameter, it is possible to see that ALR and FB-ALR show the same behavior, with an upward flow of gas greater than that of the BCR for all aeration regimes. This arises from the fact that for ALR and FB-ALR, the riser cross-section was 0.00347 m^2^, while for BCR, the section was 0.00562 m^2^. This more extensive section area implies a greater volume for the bubbles to rise in the BCR, reducing their flow speed.

In contrast to *U*_G_, a purely geometric measurement, *U*_L_ revealed that in the BCR, the liquid mass rose in a shorter time for any aeration regime (*p* < 0.05). The larger sectional area of this bioreactor allowed the bubbles to coalesce and function as a “plunger” that carried the dye at a greater speed. In ALR and FB-ALR, the smaller section in the riser created a more turbulent system for the bubbles to rise, which competed for space and did not favor dye transport.

*t*_M_ is the time required for the liquid phase to obtain uniformly distributed concentrations of solutes and cells throughout its entire length; therefore, the lower the value, the better the performance. The BCR tended to present the lowest values for the different aeration regimes. Although ALR and FB-ALR have similar architecture, with a riser/downcomer ratio of 1.64, the presence of a stainless-steel screen in the latter reduced the numerical values when aeration was 0.25, 0.50, and 0.75 vvm (*p* < 0.05), probably because it attracted part of the liquid phase that went down the downcomer. Under aeration at 1.00 vvm, the intense upward traffic may have inhibited this transport through the screen. However, it must be considered that these tests were conducted with uninoculated bioreactors. After forming a biofilm covering the scaffold, the bioreactor operates as a traditional ALR.^21^ The lower *t*_M_ values observed in the BCR are because it had a smaller upward path than those of the other bioreactors, as corroborated by the *U*_L_. The fact that the liquid load did not need to be recycled via the downcomer shortened the mixing time.

Global gas retention (ε_G_) varied between bioreactors depending on the aeration regime (*p* < 0.05) and type of bioreactor (*p* < 0.05), but with variations in hydraulic gas retention occurring only from 0.75 vvm. Observed increases in ALR and FB-ALR must be because the smaller cross-section forced the bubbles to collide and rupture, increasing gas residence time and mass transfer.^30^ Although the increase in the volume of air admitted promoted changes in ε_G_, no signs of gaseous flooding were observed; in reality, the headspaces formed in the three variations of bioreactors were large enough for bubbles formed during ascension to detach and rupture, not promoting unwanted increases in the operational volumes of the bioreactors.

Elevations in *P*_H_ simultaneously increase the gas density, the instability of larger bubbles,^31^ the gas/liquid interfacial area, and the *k*_L_a measurements.^32^ This all results in a consequent increase in the continuous availability of dissolved oxygen to the cells. In Table 2, it was impossible to observe significant increases in *P*_H_ (*p* > 0.05), mainly due to the addition of *p*_atm_ in the composition of eq 6.

The pressures measured here do not constitute operation under high pressure. Our bioreactors operated with *P*_L_, which varied from 0.1095 to 0.1101 MPa, slightly above the average atmospheric pressure in Curitiba on the day the experiment was conducted. However, even with these values, we obtained increments of *k*_L_a, diverging from the behavior pointed out by Campani et al.^33^ that *P*_H_ between 0.1000 and 0.4000 MPa is insufficient for this. Those authors used an airlift bioreactor with a different architecture from ours, which may have determined this disparity in behavior.

The *k*_L_a values generally followed FB-ALR ≥ ALR ≥ BCR, with FB-ALR > BCR for all aeration regimes. Higher values for *k*_L_a imply higher rates of supply and maintenance of molecular oxygen supply from the gas phase to the liquid phase and, consequently, to the fungal biomass cells.^34^ The reticulated baffle (screen) ensures greater fragmentation of rising air bubbles.

ALR and FB-ALR operated following a riser/downcomer distribution architecture compatible with the golden ratio, which undoubtedly influenced this system’s improved performance.^20^

As the *k*_L_a at 0.75 vvm did not differ from that obtained at 1.00 vvm (*p* = 0.0673), nor did it differ from the ALR in both aeration regimes (*p* ≥ 0.0689), it was decided to use the FB-ALR with aeration of 0.75 vvm, which combines the advantages of operating at reasonable fewer power input with same mass transfer parameters, which is the main parameter for scaling up in bioprocesses involving aerobic organisms,^35^ in addition to generating a clear supernatant.^21^

### Tests in SRILAB Operating as FB-ALR

*Rhizopus* sp. UPX271019 grew for 3 days, confluently covering the submerged area of the stainless-steel mesh. The high concentration of hyphae fragments inoculated (McFarland tube #5), which, after dilution in the CDM-broth within the bioreactor, have reached ∼2.7 × 10^5^ cfu mL^–1^ (counted in modified Neubauer reticulum), certainly guaranteed such coverage in the form of biofilm. Once this biofilm formation was complete, the SRILAB began to function as a bioreactor with characteristics of both an airlift and a fixed bed.^21,22^

Operating as FB-ALR, L-ASNase was secreted by fungal cells grown under a biofilm phenotype, which allows separation without requiring centrifugation or filtration, facilitating subsequent downstream steps.^22^ The biofilms covered the entire surface of the baffles, and no cell masses were dislodged.

Five days after adding l-asparagine, which acted as an enzymatic inducer, the enzymatic activity for crude supernatant was 2475 ± 710 U. The protein concentration (0.800 ± 0.052 μg_protein_ mL^–1^) was adopted as a divider to determine the specific enzymatic activity (3093 ± 606 U μg_protein_^–1^), which differed from the biomass used in screening assays. This practice is used in enzyme purification procedures, making monitoring their enrichment easier by increasing specific activity.^36^

Other high values for L-ASNase activity have previously been achieved (825.4 to 1992 U), but only after downstream enzyme purification using cold acetone and ion exchange chromatography,^37,38^ which were not employed in this study. To our knowledge, the most significant values for crude activity of fungal L-ASNase are those presented by our group using the zygomycete *Cunninghamella echinulata* DSM1905 grown as a biofilm in an FB-ALR, with an enzyme activity of 1173 and a specific enzyme activity of 279.22.^22^ The results obtained here reveal that *Rhizopus* sp. UPX271019 produces superior amounts of L-ASNase (2475 U) and deserves special attention.

The specific enzyme activity obtained (3,093 ± 60 U μg_protein_^–1^) indicates a high proportional fraction of L-ASNase among general extracellular proteins. This must occur due to the sum of factors that include intrinsic strain/species characteristics, the inductor use, high aeration regimen, cells grown as biofilms, etc.

As pointed out recently,^22^ the L-ASNase produced in biofilm bioreactor was consistently higher (*p* < 0.001) than in conical flasks during screening stages (12.64 ± 0.98 U), commonly employed in screening trials.^39^ Indeed, bioreactors that operate with microbial cells grown in biofilms have proven more efficient in obtaining molecules of interest.^40,41^

Hybrid bioreactor variants such as FB-ALR have proven very efficient in drug biotransformation.^21^ Although this study was conducted in a lab-scale biofilm bioreactor, it can help the industry produce therapeutic enzymes like L-ASNase. Further studies with a pilot-scale version of FB-ALR may confirm such a possibility.

## Conclusion

Based on the experimental data, it can be inferred that *Rhizopus* spp. are proficient producers of L-ASNase, yielding the enzyme in significant quantities. Furthermore, a hybrid system integrating an airlift bioreactor with a fixed-bed represents a promising approach for enzyme production. This configuration leverages the superior mass transfer capabilities of the airlift bioreactor for enhanced nutrient distribution and gas exchange. At the same time, the fixed-bed component facilitates the complete formation of a robust fungal biofilm, leading to a clear supernatant and simplified downstream processing.

Fungal-derived L-ASNases present a compelling alternative for enhancing the current anticancer therapeutic landscape. Our research efforts are directed toward addressing key challenges to realize this potential, including: the industrial scalability of the upstream bioprocess; the optimization of downstream improvements, specifically utilizing an aqueous two-phase system (ATPS) for efficient enzyme recovery and concentration; and the confirmation of reduced toxicity compared to L-ASNase derived from bacterial sources.

## References

[1] Fu Y., Ding L., Yang X., Ding Z., Huang X., Zhang L., Chen S., Hu Q., Ni Y. (2021). Asparagine
synthetase-mediated L-asparagine metabolism disorder promotes the
perineural invasion of oral squamous cell carcinoma. Front Oncol..

[2] Grima-Reyes M., Vandenberghe A., Nemazanyy I., Meola P., Paul R., Reverso-Meinietti J., Martinez-Turtos A., Nottet N., Chan W. K., Lorenzi P. L., Marchetti S., Ricci J. E., Chiche J. (2022). Tumoral microenvironment
prevents de novo asparagine biosynthesis in B cell lymphoma, regardless
of ASNS expression. Sci. Adv..

[3] Yuan Q., Yin L., He J., Zeng Q., Liang Y., Shen Y., Zu X. (2024). Metabolism
of asparagine in the physiological state and cancer. Cell Commun. Signal..

[4] Saleh A. A., El-Aref H. M., Ezzeldin A. M., Ewida R. M., Al-Bedak O. A. M. (2025). Enhanced
production and purification of L-asparaginase from *Bacillus
paralicheniformis* AUMC B-516 with potent cytotoxicity against
MCF-7 cell lines. AMB Express.

[5] MacDonald V. S., Thamm D. H., Kurzman I. D. (2005). Does L-asparaginase
influence efficacy or toxicity when added to a standard CHOP protocol
for dogs with lymphoma?. J. Vet. Int. Med..

[6] LeBlanc A. K., Cox S. K., Kirk C. A., Newman S. J., Bartges J. W., Legendre A. M. (2007). Effects of L-asparaginase
on plasma amino acid profiles
and tumor burden in cats with lymphoma. J. Vet.
Int. Med..

[7] FDA . Nomination from the American Veterinary Medical Association for “Lists of Bulk Drug Substances for Compounding: Office Stock Drugs for Use in Nonfood-Producing Animals or Drugs for Use in Food-Producing Animals or Free-Ranging Wildlife Species", FDA-2018-N-4626–0725, FDA https://www.regulations.gov/document/FDA-2018-N-4626-0725 (accessed Oct 06, 2025).

[8] Blake M. K., Carr B. J., Mauldin G. E. (2016). Hypersensitivity
reactions associated
with L-asparaginase administration in 142 dogs and 68 cats with lymphoid
malignancies: 2007–2012. Can. Vet. J..

[9] Dias F. F. G., Santos Aguilar J. G. D., Sato H. H. (2019). L-asparaginase from *Aspergillus* spp.:
production based on kinetics, thermal
stability and biochemical characterization. 3 Biotech.

[10] Thirunavukkarasu N., Suryanarayanan T. S., Murali T. S., Ravishankar J. P., Gummadi S. N. (2011). L-asparaginase from
marine derived fungal endophytes
of seaweeds. Mycosphere.

[11] Doriya K., Kumar D. S. (2016). Isolation and screening of L-asparaginase
free of glutaminase
and urease from fungal sp. 3 Biotech.

[12] Freitas M., Souza P., Cardoso S., Cruvinel K., Abrunhosa L. S., Ferreira Filho E. X., Inácio J., Pinho D. B., Pessoa A., Magalhães P. O. (2021). Filamentous
fungi producing L-asparaginase with low
glutaminase activity isolated from Brazilian savanna soil. Pharmaceutics.

[13] Waldherr P., Bliatsiou C., Böhm L., Kraume M. (2023). Fragmentation of *Aspergillus niger* pellets in stirred tank bioreactors due
to hydrodynamic stress. Chem. Eng. Res. Des..

[14] Sarquis M. I. d. M., Oliveira E. M. M., Santos A. S., Costa G. L. (2004). Production
of L-asparaginase by filamentous fungi. Mem.
Inst. Oswaldo Cruz.

[15] Souza P. M., de Freitas M. M., Cardoso S. L., Pessoa A., Guerra E. N. S., Magalhães P. O. (2017). Optimization
and purification of
L-asparaginase from fungi: A systematic review. Crit. Rev. Oncol. Hematol..

[16] da
Cunha M. C., Dos Santos Aguilar J.
G., de Melo R. R., Nagamatsu S. T., Ali F., de Castro R. J. S., Sato H. H. (2019). Fungal L-asparaginase: Strategies for production and
food applications. Food Res. Int..

[17] Garcia P. H. D., Costa-Silva T. A., Gómez M. M., Contesini F. J., Canella P. R. B. C., Carvalho P. D. O. (2023). Anticancer asparaginases:
perspectives in using filamentous fungi as cell factories. Catalysts.

[18] Osama S., El-Sherei M. M., Al-Mahdy D. A., Bishr M., Salama O., Raafat M. M. (2023). Optimization and characterization of antileukemic L-asparaginase
produced by *Fusarium solani* endophyte. AMB Express.

[19] Almeida, R. P. C. D. Avaliação da produção de L-asparaginase por fungos isolados do bioma Cerrado; PhD Dissertation, University of Brasilia 2015 https://web.archive.org/web/20180720220510id_/; http://repositorio.unb.br/bitstream/10482/18458/1/2015_RenataPaulaCoppinideAlmeida.pdf.

[20] Rosa E. A., Bianchini L. F., da Silva Ramos R. C., Arantes A. B., da Silva R. F., Glassey J. (2019). Hydrodynamics
of split-rectangle-internal loop airlift
bioreactor with variations in riser and downcomer cross-sectional
areas based on the golden ratio. J. Chem. Technol.
Biotechnol..

[21] Bianchini L. F., da Silva
Ramos R. C., de Oliveira N. S., de Paula R. C., Rosa R. T., Glassey J., Rosa E. A. (2021). Drug biotransformation process favored
by fungal biofilms formed on a proposed fixed bed-airlift hybrid reactor. J. Chem. Technol. Biotechnol..

[22] Ramos R. C. P. D. S., de Oliveira N. S., Bianchini L. F., Azevedo-Alanis L. R., Pimentel I. C., Hardy A. M. T. G., Murata R. M., Glassey J., Rosa E. A. R. (2024). *Cunninghamella
echinulata* DSM1905 biofilm-based L-asparaginase production
in pneumatically-driven bioreactors. PLoS One.

[23] Śmiech K. M., Kovács T., Wildschut R. F., Criado Monleon A. J., de Vries-Onclin B., Bowen J. G., Agostinho L. L. F. (2020). Thermal
disinfection of hospital wastewater in a pilot-scale continuous-flow
system. Appl. Water Sci..

[24] Bradford M. (1976). A rapid and
sensitive method for the quantification of microgram quantities of
protein utilizing the principle of protein-dye binding. Anal. Biochem..

[25] Ashok A., Kumar D. S. (2021). Laboratory scale bioreactor studies
on the production
of L-asparaginase using *Rhizopus microsporus* IBBL-2
and *Trichosporon asahii* IBBLA1. Biocatal. Agric. Biotechnol..

[26] Lennartsson, P. R. ; Taherzadeh, M. J. ; Edebo, L. Rhizopus. In Reference Module in Food Science. Encyclopedia of Food Microbiology, 2nd ed.; Batt, C. A. ; Tortorello, M. L. , Eds.; Elsevier Ltd, 2014; pp 284–290 10.1016/B978-0-12-384730-0.00293-1.

[27] Anchundia M., León-Revelo G., Santacruz S., Torres F. (2025). Production of β-glucans
from *Rhizopus oryzae* M10A1 by optimizing culture
conditions using liquid potato starch waste. Polymers.

[28] Jennessen J., Nielsen K. F., Houbraken J., Lyhne E. K., Schnürer J., Frisvad J. C., Samson R. A. (2005). Secondary metabolite and mycotoxin
production by the *Rhizopus microsporus* group. J. Agric. Food Chem..

[29] Meyer V., Cairns T., Barthel L., King R., Kunz P., Schmideder S., Müller H., Briesen H., Dinius A., Krull R. (2021). Understanding and controlling filamentous growth of fungal cell factories:
novel tools and opportunities for targeted morphology engineering. Fungal Biol. Biotechnol..

[30] Talaia M. A. R. (2007). Terminal
velocity of a bubble rise in a liquid column. World Acad. Sci. Eng. Technol..

[31] Wilkinson P. M., Dierendonck L. L. V. (1990). Pressure and gas density effects on bubble break-up
and gas hold-up in bubble columns. Chem. Eng.
Sci..

[32] Linek V., Benes P., Vacek V. (1989). Dynamic pressure
method for k_L_a measurement in large-scale bioreactors. Biotechnol. Bioeng..

[33] Campani G., Ribeiro M. P., Horta A. C., Giordano R. C., Badino A. C., Zangirolami T. C. (2015). Oxygen transfer in a pressurized airlift bioreactor. Bioprocess Biosyst. Eng..

[34] Romo-Buchelly R. J., Sepúlveda-Arango L. J., Restrepo-Restrepo Y. P., Areiza-Restrepo D. E., Henao S. Z., Garcés L. A. (2022). volumetric
oxygen transfer coefficient effect on biomass, bioactive compounds
production, and kinetic behavior of *G. lucidum* in
submerged culture using a complex medium. Braz.
Arch. Biol. Technol..

[35] Tajsoleiman T., Mears L., Krühne U., Gernaey K. V., Cornelissen S. (2019). An industrial
perspective on scale-down challenges using miniaturized bioreactors. Trends Biotechnol..

[36] Thakur M., Lincoln L., Niyonzima F. N., More S. S. (2014). Isolation, purification
and characterization of fungal extracellular L-asparaginase from *Mucor hiemalis*. J. Biocatal. Biotransform..

[37] Lincoln L., Niyonzima F. N., More S. S. (2015). Purification and
properties of a
fungal L-asparaginase from *Trichoderma viride* Pers:
SF GREY. J. Microbiol. Biotechnol. Food Sci..

[38] Shafei M. S., El-Refai H. A., Mostafa H., El-Refai A. M. H., El-Beih F. M., Easa S. M., Gomaa S. (2015). Purification, characterization,
and
kinetic properties of *Penicillium cyclopium* L-asparaginase:
impact of lasparaginase on acrylamide content in potato products and
its cytotoxic activity. Curr. Trends Biotechnol.
Pharm..

[39] de
Oliveira N. S., da Silva G., P L., Furlan O., Peña L. C., Bianchini L. F., Parahitiyawa N., Rosa E. A. R. (2024). The song remains
the same. The lab bench dilemma of using shaken flasks in microbial
biotransformation experiments. Biocatal. Biotransform..

[40] Muffler K., Lakatos M., Schlegel C., Strieth D., Kuhne S., Ulber R. (2014). Application of biofilm
bioreactors in white biotechnology. Adv. Biochem.
Eng. Biotechnol.

[41] Carvalho F. M., Azevedo A., Ferreira M. M., Mergulhão F. J. M., Gomes L. C. (2022). Advances on bacterial
and fungal biofilms for the production
of added-value compounds. Biology.

